# Notes on Brain Fever

**Published:** 1908-10-10

**Authors:** 


### The Practitioner's Relaxations.

### NOTES ON BRAIN FEVER.

As a recreation from other more strenuous pursuits, and as a pastime for the hotter part of Indian hot-weather days, I have made a little excursion into a by-way of medicine not hitherto wandered in by the authors of text-books. For, so far as I can ascertain, brain fever is, as yet, not scientifically described, nor do I find it in the official Nomenclature of Diseases.

It is a strange disease, very common—in novels. Comparatively recently, that is within the last fifty years, the number of cases has vastly increased, till by now many thousands are recorded. This might seem to make the disease a menace to the public; but it is not to be feared by the general public. It appears only to affect the population that dwells in books and it appears only to affect heroes and heroines. Villains may take comfort; they seldom are afflicted. The common herd may be happy; they never are.

No notes have been kept of the ætiology of the disease. This is accounted for by the fact that no medical man has described it. There is no record of a negro or a Chinaman being attacked; in fact, the disease is confined to a small section of the white races of the world. Amongst that small section it is astonishly frequent. I reckon about ten per cent. of heroes and heroines have an attack. Nor is one attack protective against a second. A hero may certainly have two attacks in a twelvemonth, because I have seen the second. And a relapse, one to two days after apparently complete recovery, is not uncommon, and always convenient —to the author. Though the ætiology is unknown, the exciting cause is fairly definite. There must be trouble and worry, or a shock. A jilted heroine is always in danger; so is a misunderstood hero. It is in fact almost criminal to misunderstand a hero, especially if he has done all he knows to be misunderstood, for it is so likely to bring on an attack. The diagnosis is apparently easy. No doubt is ever expressed on the subject, though sometimes the symptoms might possibly be mistaken for hysteria, drink, or lunacy. They never are so mistaken, because heroes and heroines do not suffer from hysteria or from drink. A hero may temporarily give way to drink whilst waiting for a heroine to cease from misunderstanding him. Both may suffer from insanity, but it is always melancholic in character, and temporary.

However, as a fact there is never any difficulty in the diagnosis. Any hospital nurse who happens to be present at the first onset of the attack will spot the symptoms like a shot. "You had better send for a doctor," she will say, "it looks very like brain fever coming on."

What are the symptoms? After trouble the heroine suddenly begins to make a speech, preceded and punctuated by sobs. Her hands are usually "burning hot"; her cheeks are "flushed"; she often "trembles in every limb." Hence we may presume a rise of temperature, though no charts have been recorded. She however will continue her speech, and "stare with bright unseeing eyes." After a speech of about five hundred words she frequently "clutches at her throat" for "something seemed to rise there and threaten to suffocate her." This symptom seems akin to the globus hystericus. Then she "bursts into a flood of hysterical tears" and "sinks"—it is remarkable that she never flops ungracefully—"on the floor, and becomes insensible." After this, "delirium supervenes," she "tosses on her bed."

The total duration of the attack is usually fourteen whole days. It is during the period that succeeds the sudden collapse and insensibility that this strange disease is really most interesting. Though the preliminary stages may differ in different cases, this stage of delirium is always marked by long statements made by the delirious patient. No muttering delirium with picking at the bed clothes, and here and there a word. Instead, long connected grammatical sentences; often uttered somewhat jerkily, but still the grand whole perfectly intelligible. Heroes and heroines, when their brains are acting normally, often manage to hash up their affairs; but give them brain fever, and they will talk straight on end for fourteen days and nights, and clear up the very worst tangles they have got themselves into.

There is no pathological anatomy of these cases, and the reason is not far to seek; the prognosis is invariably favourable. No fatal case has yet been recorded. The good doctor always restores her to life. He might allay the anxiety of the relatives by explaining how harmless it really is. But the good doctor is worldly wise. He gives a grave prognosis, and takes credit for the favourable result.

How does he obtain this favourable result? That also is not made clear. Frequently he gives an opiate but, as the patient in spite of the opiate never ceases to talk till he or she has had her say out, that does not seem to be the curative agent. Often he "shears off her masses of nut-brown hair," and applies ice-bags. But on the whole he doesn't do much. This is wise, for he knows the case will end well, and besides he seldom has more than one nurse, usually an amateur, who watches "day and night with unremitting attention and care," and who is not so knocked up at the end of a fortnight as a mere ordinary trained nurse would be.

The amateur nurses who nurse brain fever cases are a truly remarkable set of women. Often they are under twenty-one years of age, and have "led a butterfly existence." Sometimes they are older, and have been chronic invalids surrounded by soft things and flowers all the earlier part of the book. Yet quite without training they do day and night nursing for a fortnight on end, eating their meals by the patient's bed and performing all their duties with the greatest ease and success.

